# Prediction of Distal Dural Ring Location in Internal Carotid Paraclinoid Aneurysms Using the Tuberculum Sellae–Anterior Clinoid Process Line

**DOI:** 10.3390/jcm14175951

**Published:** 2025-08-22

**Authors:** Masaki Matsumoto, Tohru Mizutani, Tatsuya Sugiyama, Kenji Sumi, Shintaro Arai, Yoichi Morofuji

**Affiliations:** 1 Department of Neurosurgery, Showa Medical University, Tokyo 142-8555, Japan; sumiken202@med.showa-u.ac.jp (K.S.);; 2 Department of Neurosurgery, Showa Medical University Koto Toyosu Hospital, Tokyo 135-8577, Japan; mizutani.nsutky@gmail.com (T.M.); tasugiyama@med.showa-u.ac.jp (T.S.)

**Keywords:** distal dural ring, paraclinoid aneurysm, tuberculum sellae, anterior clinoid process

## Abstract

**Background/Objectives**: Current bone-based landmark approaches have shown variable accuracy and poor reproducibility. We validated a two-point “tuberculum sellae–anterior clinoid process” (TS–ACP) line traced on routine 3D-computed tomography angiography (CTA) for predicting distal dural ring (DDR) position and quantified the interobserver agreement. **Methods**: We retrospectively reviewed data from 85 patients (87 aneurysms) who were treated via clipping between June 2012 and December 2024. Two blinded neurosurgeons classified each aneurysm as extradural, intradural, or straddling the TS–ACP line. The intraoperative DDR inspection served as the reference standard. Diagnostic accuracy, χ^2^ statistics, and Cohen’s κ were calculated. **Results**: The TS–ACP line landmarks were identifiable in all cases. The TS–ACP line classification correlated strongly with operative findings (χ^2^ = 138.3, *p* = 6.4 × 10^−29^). The overall accuracy was 89.7% (78/87), and sensitivity and specificity for identifying intradural aneurysms were 94% and 82%, respectively. The interobserver agreement was substantial (κ = 0.78). Nine aneurysms were misclassified, including four cavernous-sinus lesions that partially crossed the DDR. Retrospective fusion using constructive interference in steady-state magnetic resonance imaging corrected these errors. **Conclusions**: The TS–ACP line represents a rapid, reproducible tool that reliably localizes the DDR on standard 3D-CTA, showing higher accuracy than previously reported single-landmark techniques. Its high accuracy and substantial inter-observer concordance support incorporation into routine preoperative assessments. Because the method depends on only two easily detectable bony points, it is well-suited for automated implementation, offering a practical pathway toward artificial intelligence-assisted stratification of paraclinoid aneurysms.

## 1. Introduction

Internal carotid artery (ICA) paraclinoid aneurysms represent 5–11% of all intracranial aneurysms and present significant diagnostic and therapeutic challenges [1]. The critical factor when determining appropriate management strategies for such aneurysms is the aneurysm’s relationship to the distal dural ring (DDR). This structure separates the intradural space, which carries a substantial risk of subarachnoid hemorrhage (SAH) rupture, from the extradural cavernous sinus, where aneurysms rarely rupture [2]. Despite its clinical importance, the DDR cannot be directly visualized using conventional computed tomography angiography (CTA) or magnetic resonance angiography (MRA), thereby necessitating indirect prediction methods based on anatomical landmarks to be identified [3,4]. Current approaches based on bony landmarks have shown variable levels of accuracy in this regard, with significant inter-observer disagreement limiting their clinical reliability [5,6,7,8].

In addition to the risk of rupture, paraclinoid aneurysms frequently produce neuro-ophthalmic symptoms—most commonly visual field deficits—because of the intimate relationship between the carotid ophthalmic segment and the optic apparatus. A single-center cohort analyzing flow diverter procedures reported visual symptoms in 25% of treated cases and identified aneurysm enlargement and early ophthalmic artery compromise as modifiable risk factors [9]. Such data highlight the need for accurate classification of the location of paraclinoid aneurysms before intervention.

Therapeutically, the management landscape has shifted toward flow diversion for large or morphologically complex paraclinoid aneurysms [10]. A 2024 meta-analysis of 1680 unruptured aneurysms treated with the surface-modified Pipeline Shield device reported 79% complete occlusion at 12 months with <2% major ischemic events [11]. Next-generation devices, such as the new flow diverter evaluated in a 2025 multicenter study across seven hospitals, have demonstrated 81% complete occlusion and no device-related mortality [12].

However, while advances in endovascular technology have broadened therapeutic options, the ability to distinguish between intradural and extradural lesions remains a fundamental prerequisite for safe and effective treatment selection. In clinical practice, this distinction frequently hinges on imaging surrogates of the DDR, given its invisibility on conventional CTA and MRA. Therefore, there is a pressing need for a simple, reproducible method to approximate the DDR’s location with high reliability.

The tuberculum sellae (TS) and anterior clinoid process (ACP) represent anatomical landmarks that are consistently visible via 3D-CTA. Based on the findings of detailed anatomical studies that have confirmed the predictable relationship between these structures and the DDR, we hypothesized that a virtual line connecting the TS and ACP could be used to accurately predict the DDR’s location. Therefore, this study aimed to validate the diagnostic accuracy and inter-rater reliability of the TS–ACP line method on 3D-CTA for paraclinoid aneurysms, using direct intraoperative findings as the gold standard, and establish its utility as a practical tool in daily clinical practice.

## 2. Materials and Methods

### 2.1. Study Design and Patient Selection

We performed a retrospective analysis of data from 85 patients, with 87 total aneurysms, who underwent craniotomy clipping to treat paraclinoid aneurysms at Showa Medical University Hospital between June 2012 and December 2024. Preoperative 3D-CTA was performed in all patients. The hospital’s institutional review board (IRB) approved the study’s protocol (approval no.: 21-007-A). Patient consent was obtained under an opt-out policy approved by the IRB. A public notice describing the study and the option to decline participation was posted on the hospital website. Written informed consent was waived due to the retrospective design and anonymization.

### 2.2. Surgical Procedure

A standard frontotemporal craniotomy was utilized in all cases. An extradural anterior clinoidectomy was performed to expose the paraclinoid region and facilitate safe aneurysm clipping. Following the dural opening, the senior surgeon meticulously examined the relationship between the aneurysm neck and dome and the DDR.

### 2.3. 3D-CTA Acquisition

CTA was performed using either a 64-section SOMATOM Sensation scanner (Siemens Healthineers, Erlangen, Germany) or a dual-source 2 × 128-section SOMATOM Force scanner (Siemens Healthineers, Erlangen, Germany). A standard volume of non-ionic iodinated contrast material was administered intravenously, with image acquisition timed using a bolus-tracking technique in the cervical ICA.

The imaging parameters were as follows, with auto-exposure control (CARE Dose) enabled:

- SOMATOM Sensation: Collimation: 0.6 mm, tube voltage: 120 kVp, tube current: 240 mA, rotation time: 0.5 s.
- SOMATOM Force: Collimation: 0.6 mm, tube voltage: 90 kVp, quality reference mAs: 230 mA, pitch factor: 1.2, rotation time: 0.5 s.

Axial source images were reconstructed with a slice thickness of 0.6 mm and transferred to a dedicated workstation for analysis.

### 2.4. TS–ACP Line Method

**Figure 1:**
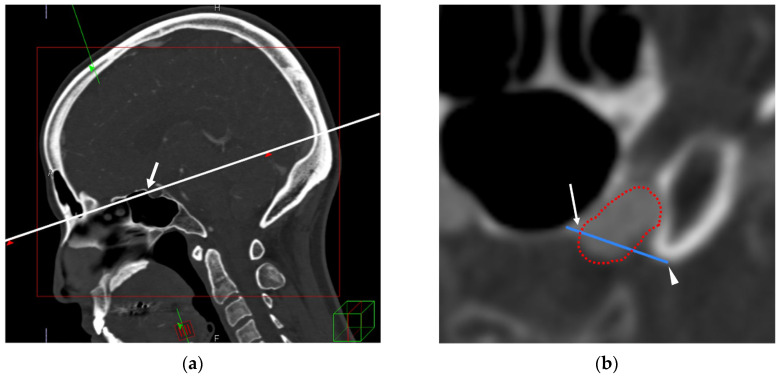
(**a**) Multiplanar reconstruction aligned to visualize the TS (arrow) and the ACP in the same plane (white line). (**b**) On the plane defined in (**a**), the TS–ACP line (blue line) is defined as the straight line connecting the point where the upper edge of the TS meets the ICA (arrow) to the tip of the ACP (arrowhead). The red dotted line represents the ICA. TS–ACP: tuberculum sellae–anterior clinoid process; ICA: internal carotid artery.

Using multiplanar reconstruction on a Ziostation workstation (Ziosoft, Inc., Tokyo, Japan), we first adjusted the viewing planes to ensure that the TS and the tip of the ACP were visualized in the same plane. The TS–ACP line was subsequently defined as the straight line connecting the point at which the upper edge of the TS meets the ICA and the tip of the ACP (Figure 1).

**Figure 2:**
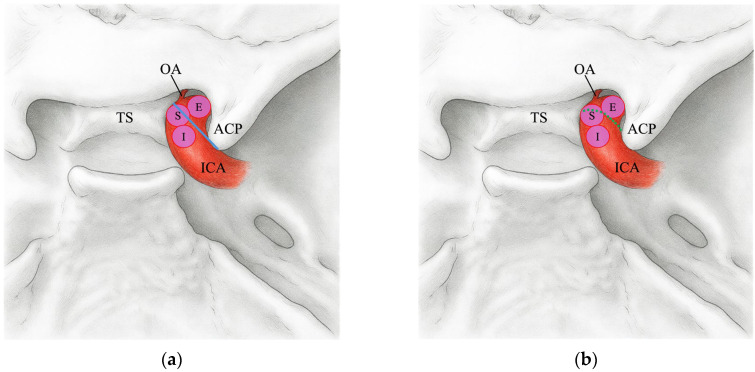
Schematic of paraclinoid aneurysm positions. (**a**) Positions of extradural (E), intradural (I), and straddle (S) aneurysms relative to the TS–ACP line (blue line). (**b**) The same aneurysm positions relative to the DDR (green dotted line). OA: ophthalmic artery, TS: tuberculum sellae, ACP: anterior clinoid process, ICA: internal carotid artery.

Subsequently, we classified the aneurysm domes that were proximal to the TS–ACP line on preoperative 3D-CTA as extradural (E), those that were distal to the TS–ACP line as intradural (I), and those that straddled the line as straddle (S). We also categorized the positional relationship between the aneurysm dome and the DDR as observed intraoperatively into three classifications, and compared these findings with those assessed using the TS–ACP line (Figure 2).

### 2.5. Image Analysis

Two trained neurosurgeons who were blinded to the clinical patient information and reference standard results independently analyzed all of the cases.

### 2.6. Statistical Analysis

Categorical variables, including preoperative the TS–ACP line classifications and intraoperative DDR categories, were summarized as frequencies and percentages. Their associations were assessed using a 3 × 3 contingency table and Pearson’s chi-squared test, with statistical significance set at a two-tailed *p* value of <0.05. Inter-method agreement was quantified using Cohen’s κ, interpreted according to Landis and Koch (κ = 0.61–0.80 meaning substantial, κ = 0.81–1.00 meaning almost perfect). The overall predictive accuracy (correct predictions divided by the total number of cases) was calculated to convey the clinical utility of the approach. All statistical computations were performed using JMP Pro 17 (SAS Institute Inc., Cary, NC, USA).

## 3. Results

This study included 85 patients (mean age: 51 years; 14 males and 71 females) with a total of 87 paraclinoid aneurysms. The mean aneurysm size was 6.8 ± 2.8 mm (range, 3–18 mm). Table 1 summarizes the cross-classification between the preoperative TS–ACP line assessments and intraoperatively determined DDR location.

**Figure 3:**
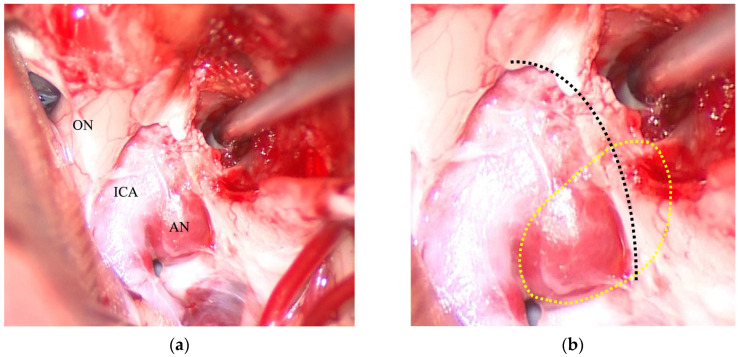
Intraoperative findings of a representative discordant case classified as TS–ACP–I but confirmed as DDR–S: views after anterior clinoidectomy and the DDR opening. (**a**) After clinoidectomy and incision of the DDR, the optic nerve (ON), internal carotid artery (ICA), and aneurysm (AN) are exposed, demonstrating intra- and extradural components. (**b**) Magnified view of the same field. The aneurysm dome (yellow dotted outline) straddles the DDR, which is traced by the black dotted line. TS–ACP: tuberculum sellae–anterior clinoid process; DDR: distal dural ring.

The association between the two classifications was found to be highly significant (χ^2^ = 138.3, *p* = 6.4 × 10^−29^). An agreement analysis yielded a Cohen’s κ of 0.78, indicating substantial concordance. Overall, 78 of the 87 aneurysms (89.7%) were correctly classified using the TS–ACP line, with a 95% confidence interval (CI) of 81.5–94.5%. Column-wise sensitivity by intraoperative DDR category was 100.0% (95% CI, 20.7–100.0%), 91.2% (81.1–96.2%), and 86.2% (69.4–94.5%) for DDR-E, DDR-I, and DDR-S, respectively. Row-wise precision (positive predictive value) by the TS–ACP line category was 100.0% (20.7–100.0%), 92.9% (83.8–97.2%), and 83.3% (66.4–92.7%) for line-E, line-I, and line-S, respectively. There were nine cases where the preoperative assessment did not match the intraoperative findings. Of particular note were four cases that were predicted to be the TS–ACP line-I but confirmed to be DDR-S intraoperatively. All of these were cavernous sinus aneurysms (Figure 3). Given the relatively small extradural (*n* = 1) and straddle (*n* = 5) subgroup sizes, these subgroup estimates are imprecise and should be cautiously interpreted.

## 4. Discussion

This study validated the TS–ACP line as a reliable method for predicting the DDR locations of paraclinoid aneurysms, demonstrating its substantial inter-rater agreement (κ = 0.78) and high diagnostic accuracy (89.7%). These results compare favorably with existing landmark-based methods, while offering practical advantages for routine clinical implementation.

### 4.1. Anatomical Basis and Technical Considerations

The TS–ACP line exploits the consistent anatomical relationship between these bony landmarks and the DDR. Microanatomical studies have confirmed that the DDR attaches laterally near the ACP base, while extending medially toward the diaphragmatic sellae above the TS [13,14,15]. Our geometric approach approximates this oblique course and provides a reproducible reference plane. The substantial inter-rater agreement (κ = 0.78) surpasses previously reported values for TS (κ = 0.138) and approaches that of optic strut landmarks (κ = 0.462) [5]. This improvement likely reflects the reliance of the method on two anatomical points rather than on single landmarks, thereby reducing the variability between interpretations.

### 4.2. Clinical Implications

**Figure 4:**
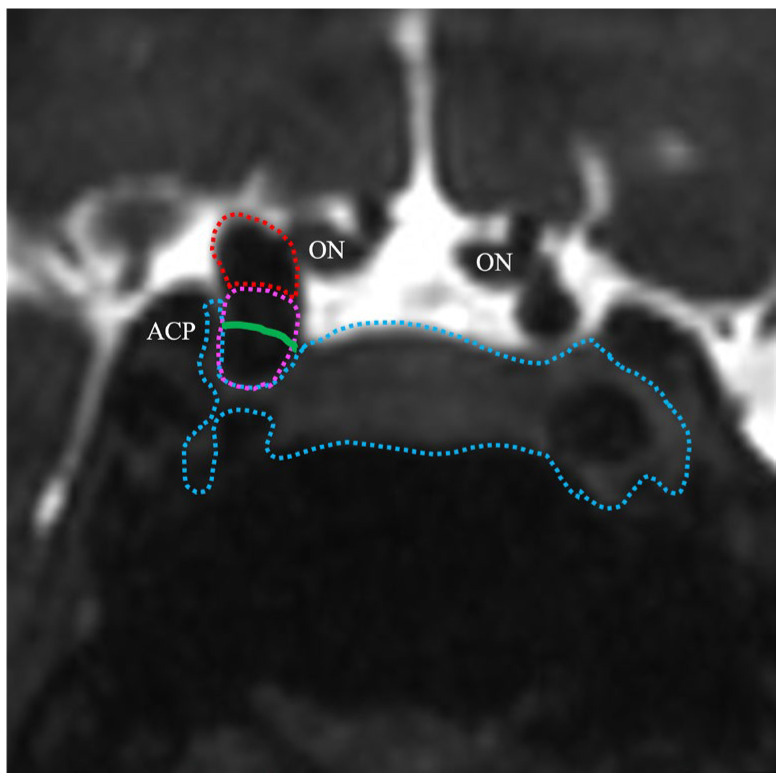
CISS MRI image of a representative discordant case classified as TS–ACP line–I but confirmed as DDR–S. CISS shows the parasellar anatomy: the ICA (red dotted outline), the aneurysm (pink dotted outline), and the cavernous sinus (blue dotted outline); the green line indicates the DDR. ON: optic nerve, ACP: anterior clinoid process, DDR: distal dural ring, CISS MRI: constructive interference in steady state magnetic resonance imaging, TS: tuberculum sellae.

Accurate DDR localization fundamentally affects treatment decisions. Intradural aneurysms carry the risk of SAH rupture, thus necessitating interventions. Conversely, extradural lesions frequently warrant nothing more than conservative management unless they are symptomatic [2]. Transitional aneurysms that straddle the DDR present particular challenges. Our 86.2% accuracy for identifying these lesions, although lower than that for purely intradural or extradural aneurysms, represents a clinically acceptable level of performance given their inherent complexity. Integration with constructive interference in steady state magnetic resonance imaging (CISS MRI) data has significantly improved transitional aneurysm classification, suggesting a complementary multimodal approach for ambiguous cases [16,17]. Preoperative high-resolution CISS MRI was performed in all of our patients. A retrospective review of the four discordant cases that were classified as TS–ACP-I but found intraoperatively to be DDR-S revealed that, in each instance, ~50% of the aneurysm dome was embedded within the cavernous sinus (Figure 4).

### 4.3. Comparison with Alternative Methods

The TS–ACP line performed better than all single-landmark-based methods in our comparative analysis. The four-point DDR plane method proposed by Scerbak et al. reported 100% correlation; however, it required the identification of multiple landmarks that were only visible in 82–89% of the cases the authors analyzed [4]. Our two-point method achieved landmark visualization in 100% of our cases, while maintaining a high level of accuracy. Cheng et al. used a virtual line connecting the inferior border of the ACP to the TS; however, they used it to identify the proximal dural ring rather than the DDR [18]. In our study, the TS–ACP line is explicitly defined by connecting the TS and the tip of the ACP and is used to predict the location of the DDR, which forms the true anatomical boundary between the intradural subarachnoid space and the extradural cavernous sinus. The proximal dural ring does not coincide with the intradural–extradural transition of the ICA. Accordingly, these are anatomically distinct structures; the proximal dural ring is an inappropriate surrogate for determining whether a paraclinoid aneurysm is located intra- or extradurally [19]. Although we previously proposed predicting the DDR by referencing the falciform ligament, the TS–ACP line introduced herein is even more practical because it can be applied directly to routine 3D-CTA datasets without the requirement for specialized imaging protocols or additional postprocessing [20]. While our approach relies on a line constructed from routine 3D-CTA, contemporary MRI techniques can visualize peri-sellar membranes and the DDR more directly. Early MRI approaches—including 1.5 T fusion MR cisternography/time-of-flight-MRA and high resolution T2 turbo spine echo technique at 3 T MRI—have been proven to be feasible, but only in small single-center samples with scant or absent surgical verification and no data concerning treatment impact. Therefore, their accuracy and clinical utility remain uncertain [21,22]. Similarly, Oki et al. reported reliable DDR visualization with a novel MRI sequence; however, all paraclinoid aneurysms in their cohort were treated exclusively by endovascular means; therefore, no intra-operative benchmark was available to validate their imaging-based classifications [23]. Contrast-enhanced 3D-CISS has been shown to differentiate paraclinoid from cavernous sinus aneurysms against surgical reference standards, whereas high-resolution 3-T T2-weighted sequences can depict the DDR and classify lesions as intra-, extra-, or transitional relative to the ring [17]. These MRI methods need specialized sequences, longer processing, and expert interpretation; thus, they are not routinely used in standard preoperative assessments [24,25]. By contrast, the TS–ACP line is derived from standard 3D-CTA available in most paraclinoid workflows and enables rapid, reproducible classification within minutes. In ambiguous cases—particularly when transitional anatomy or cavernous involvement is suspected—MRI (3D-CISS or 3-T T2-weighted protocols) can serve as a complementary problem-solving tool.

### 4.4. Limitations

This study had some limitations that merit consideration. First, its retrospective design means that the presence of selection bias cannot be fully excluded. The overall sample size was modest (*n* = 87), and the extradural subgroup comprised only a single case, thereby limiting the statistical power of the approach for that category. Notably, the single extradural aneurysm in our series was one of the clinical observations that motivated this study. Preoperatively, we thought part of the lesion was intradural; however, intraoperative inspection showed that the aneurysm was actually extradural and closely positioned near the DDR. Following this case, we began to apply the TS–ACP line systematically for preoperative localization. Consequently, lesions prospectively judged to be extradural were generally managed conservatively and were not operated on, which explains why only one extradural case appears in this operative series. This practice pattern results in under-representation of extradural aneurysms and imprecise subgroup estimates—an inherent limitation of any study based primarily on surgically verified cases. Therefore, we interpret the extradural subgroup with caution. To evaluate performance in extradural cases, prospective studies that deliberately include more such cases are needed. We also note that intraoperative confirmation will often be unavailable because many extradural aneurysms are managed conservatively. Additionally, at our institution, CISS imaging is available only at 1.5 T, and neither 3 T nor 3D-CISS protocols were performed during the study period. Consequently, a head-to-head comparison between the TS–ACP line and contemporary high-resolution MRI techniques (e.g., 3 T T2-weighted/TSE or 3D-CISS) was not feasible and should be addressed in future prospective studies.

### 4.5. Future Directions

The simplicity and geometric nature of the TS–ACP line make it an ideal candidate for automation. Although artificial intelligence (AI) for intracranial aneurysm assessment has advanced recently, many tools still face challenges with complex feature recognition or require large, meticulously annotated datasets [26,27,28]. We did not develop or validate an automated pipeline in this study; therefore, we present automation as a prospective direction rather than a current capability. In principle, the task reduces to the reliable identification of two robust bony landmarks (TS and ACP) on routine 3D-CTA, followed by rule-based construction of the connecting line. Given the simplicity of the two-point geometry, bony landmark detection appears well-suited to automation. Nevertheless, in this study, it remains a prospective direction, not a validated capability.

## 5. Conclusions

The TS–ACP line is a simple geometric construct that offers reliable and reproducible localization of the DDR on routine 3-D CTA. It shows higher inter-observer agreement than single-landmark methods, and it is easier to implement than multi-landmark approaches. When integrated with high-resolution CISS MRI, diagnostic performance for transitional aneurysms improves further without appreciable increases in scan time or cost, balancing accuracy with clinical efficiency.

The main limitation of this study is its retrospective design. Nonetheless, the reliance on two stable bony landmarks makes the TS–ACP line amenable to AI-driven automation. Future development of deep-learning tools to identify these landmarks could standardize preoperative assessment, reduce diagnostic variability, and ultimately support safer, more consistent clinical decision-making across diverse practice settings.

## Figures and Tables

**Table 1:** Cross-classification of preoperative TS–ACP line assessments and intraoperative DDR location.

	DDR–E	DDR–I	DDR–S
TS–ACP line–E	1	0	0
TS–ACP line–I	0	52	4
TS–ACP line–S	0	5	25

E: extradural; I: intradural; S: straddle; TS–ACP: tuberculum sellae–anterior clinoid process; DDR: distal dural ring. Accuracy metrics are reported with two-sided 95% confidence intervals calculated using the Wilson method. Subgroup estimates for extradural (*n* = 1) and straddle (*n* = 5) categories are based on relatively small samples.

## Data Availability

The data presented in this study are available upon request from the corresponding author due to institutional and ethical restrictions related to patient confidentiality.

## Abbreviations

The following abbreviations are used in this manuscript:

ICA	Internal carotid artery
DDR	Distal dural ring
TS–ACP	Tuberculum sellae–anterior clinoid process
3D-CTA	3D-computed tomography angiography
SAH	Subarachnoid hemorrhage
MRA	Magnetic resonance angiography
TS	Tuberculum sellae
ACP	Anterior clinoid process
MRI	Magnetic resonance imaging
CISS	Constructive interference in steady state
AI	Artificial intelligence
CI	Confidence interval
